# Restoration of daptomycin sensitivity with adjunctive cefazolin is associated with C-terminal MprF mutations in MRSA bacteremia isolates

**DOI:** 10.1128/aac.01547-25

**Published:** 2026-04-27

**Authors:** Kathleen P. Davis, Husain Poonawala, Connor Murphy, Cheleste Thorpe, Adriana Rosato, Bree B. Aldridge

**Affiliations:** 1 Stuart B. Levy Center for Integrated Management of Antimicrobial Resistance, Boston, Massachusetts, USA; 2 Department of Molecular Biology and Microbiology, Tufts University School of Medicine 12261, Boston, Massachusetts, USA; 3 Division of Geographic Medicine and Infectious Diseases, Department of Medicine, Tufts Medical Center 1867, Boston, Massachusetts, USA; 4 Department of Pathology and Laboratory Medicine, Tufts Medical Center 1867, Boston, Massachusetts, USA; 5 Maine Health Institute for Research, Scarborough, Maine, USA; 6 Department of Biomedical Engineering, Tufts University School of Engineering 98044, Medford, Massachusetts, USA; The Peter Doherty Institute for Infection and Immunity, Melbourne, Victoria, Australia

**Keywords:** multiple peptide resistance factor (MprF), MRSA, vancomycin, cefazolin, daptomycin

## Abstract

Daptomycin (DAP) or vancomycin (VAN) resistance can result in methicillin-resistant *Staphylococcus aureus* (MRSA) bacteremia treatment failure. Clinical trials have not yielded a clear approach to MRSA combination therapy. Furthermore, there is not a protocol approved by the Clinical Laboratory Standards Institute (CLSI) for testing most possible antibiotic combinations, and methodology used in basic research laboratories for combination testing presents significant hurdles for implementation in clinical laboratories. In response to these challenges, we developed a high-throughput antibiotic combination testing assay to measure the minimum antibiotic concentration(s) required to reach 99% growth inhibition (IC99) and used it to identify antibiotics that can be paired with DAP to restore the DAP susceptibility of DAP-resistant (DAP-R) MRSA. The IC99 recapitulated time-kill curve results and were consistent with results of single drug MIC measurements performed under CLSI conditions. Using this assay, we found that cefazolin (CFZ) restored DAP susceptibility for DAP-R MRSA with L826F and Q692E mutations in the C-terminal region of the synthase domain of the multiple peptide resistance factor MprF. CFZ also improved VAN susceptibility when used in combination with VAN against these isolates. We established the association between the L826F MprF mutation and CFZ improvement in DAP (and VAN) susceptibility using an MprF deletion strain and transcomplementation with WT MprF compared with L826F MprF. This study highlights the IC99 assay’s potential for identifying antibiotic treatment for DAP-R MRSA bacteremia and suggests a probable link between MprF synthase domain mutations and CFZ’s ability to improve or restore DAP or VAN susceptibility.

## INTRODUCTION

Methicillin-resistant *Staphylococcus aureus* (MRSA) bacteremia is associated with a 13% 7-day mortality that increases to 37.4% at 90 days (1). Persistent MRSA bacteremia is associated with a significantly higher 30-day mortality (2). In cases of persistent MRSA bacteremia where combination therapy is considered, ceftaroline (CPT) is frequently added to daptomycin (DAP) (3, 4). Thus far, clinical studies of DAP combinations (especially prospective trials) have been mostly inconclusive (5–7). These trials have taken a “one size fits all” approach and have not accounted for strain variation in response to drug combinations, which may limit the pool of patients who would benefit.

DAP resistance (8–10) and vancomycin (VAN) heteroresistance (11, 12) contribute to treatment failure in MRSA bacteremia (13). Given the slow and expensive pipeline for new antibiotic development (14–16), the investigation into combination therapy has increased, with particular focus on adjunctive β-lactams (17–19). This focus is largely due to efforts to harness the seesaw effect, a concomitant increase in β-lactam susceptibility observed with decreases in DAP or VAN susceptibility (20–23). Exactly how DAP insertion into the cell membrane leads to cell death has been the subject of much study and debate, but there is support for mechanisms involving inhibition of peptidoglycan or phospholipid biosynthesis, with membrane depolarization occurring as part of the process (24). Mutations linked to decreased susceptibility, and in some cases treatment failure, for DAP as well as VAN, have been found in a variety of key proteins involved in membrane and cell wall biosynthesis and regulation (25–27). These include the multiple peptide resistance factor MprF, a bifunctional membrane protein consisting of a synthase domain that modifies peptidoglycan (PG) with a lysine or alanine, a flippase domain that moves the resulting Lys-PG or Ala-PG to the outer leaflet of the membrane, and a central bifunctional domain that bridges the synthase and flippase domains (28, 29). A variety of point mutations across all three domains have been associated with decreased DAP and/or VAN susceptibility (28, 29). The majority of these SNPs are associated with enhanced Lys-PG synthesis and/or translocation in conjunction with the DAP-R phenotype, and they are located within what are known as “hot spot” regions in the MprF central bifunctional region and the C-terminal region (30–33).

## RESULTS

### CFZ can re-sensitize MRSA bacteremia isolates to DAP

We performed time-kill curves with DAP in pairwise combination with CPT, CFZ, linezolid (LZD), doxycycline (DOX), and trimethoprim-sulfamethoxazole (SXT) to identify antibiotics that re-sensitize MRSA isolates to DAP. We used three previously characterized DAP-R isolates: CB5012, CB1634, and CB5036 (19, 20, 22, 34–36) from three patients who failed DAP treatment for MRSA bacteremia. For each patient, isogenic DAP-sensitive (DAP-S)/DAP-resistant (DAP-R) isolate pairs were collected before and after DAP treatment (34). Antibiotic concentrations were chosen using Clinical Laboratory Standards Institute (CLSI) breakpoints to reflect clinically relevant dosing, and growth conditions were chosen based on CLSI MIC testing protocol (37) (see Materials and Methods). CLSI has not established a CFZ sensitivity breakpoint for *S. aureus*, so a conservative concentration of 2 µg/mL was chosen. This decision was based on the 2 µg/mL CLSI sensitivity breakpoint for Enterobacterales (38), as well as previous MRSA studies that have used the concentration corresponding to the value of the serum-free average concentration (9.5 µg/mL) (39) obtained for standard dosing (between 1 and 2g IV q8h) (40). Regimens that achieved ≥3 log_10_ CFU/mL decrease from the starting inoculum at 24 h were considered bactericidal, and combinations that achieved ≥2 log_10_ CFU/mL decrease compared with the most active single agent were considered synergistic (41).

**Fig 1:**
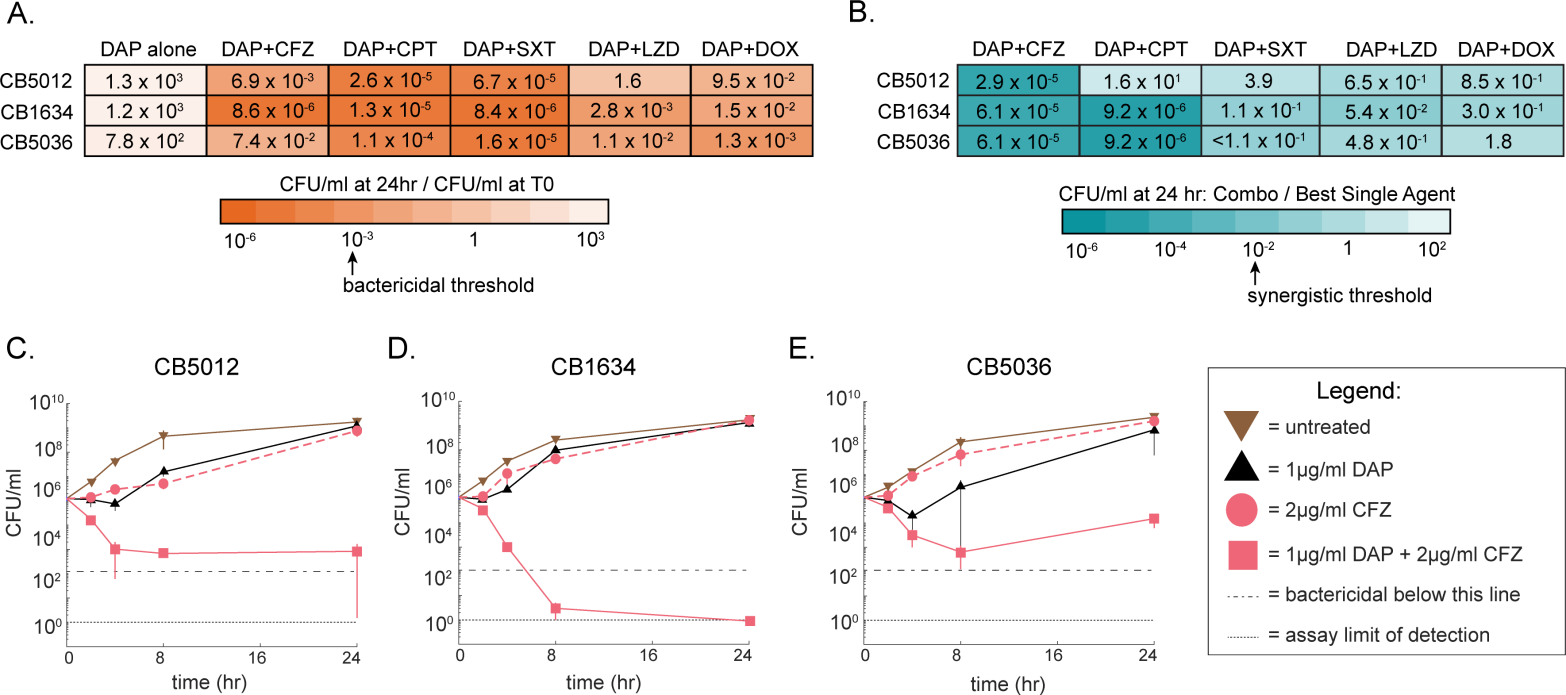
DAP + CFZ is synergistic and can be very effective at killing DAP-R MRSA isolates. (**A**) The mean of CFU/mL at 24 h divided starting CFU/mL for time-kill curves for DAP alone (at 1 µg/mL) and in pairwise combination with CFZ (at 2 µg/mL), CPT (at 1 µg/mL), SXT (at 2/38 µg/mL trimethoprim-sulfamethoxazole), LZD (at 4 µg/mL), and DOX (at 4 µg/mL), tested against DAP-R MRSA isolates CB5012, CB1634, and CB5036. The criteria for bactericidality is indicated on the color bar (darker indicates more bactericidal activity). (**B**) The mean of combination CFU/mL divided by most active constituent agent CFU/mL at 24 h for each of the same set of time-kill curves described in panel **A**. The criteria for synergy are indicated on the color bar (darker indicates more synergistic). The full-time kill curves for DAP alone, CFZ alone, and DAP+CFZ, compared with untreated control, are shown for CB5012 (**C**), CB1634 (**D**), and CB5036 (**E**). The full-time kill curves for DAP in pairwise combination with CPT, SXT, LZD, and DOX (as well as those adjunctive antibiotics used alone) tested against CB5012, CB1634, and CB5036 are shown in Fig. S1.

The only combinations that were bactericidal and synergistic against at least one isolate were DAP + CPT and DAP + CFZ. DAP + CPT is synergistic for CB1634 and CB5036 (Fig. 1A; Fig. S1) and bactericidal for all three isolates (Fig. 1B; Fig. S1). On the other hand, DAP + CFZ was synergistic for all isolates, bactericidal for all replicates of CB1634, and for two of three replicates for CB5012 (Fig. 1A through E). No other combinations were synergistic (Fig. S1). Thus, CFZ was able to re-sensitize MRSA isolates to DAP; in the case of CB1634, CFZ performed as well as CPT. These findings are consistent with prior results in which DAP + CFZ performed better than most other combinations tested against a panel of mostly DAP-S MRSA isolates (42), and support the rationale for further investigation.

It is also important to note that there was a heterogeneous response to CPT among the three isolates, as well as to CPT + DAP and CFZ + DAP, highlighting strain-to-strain variability in drug response and supporting the concept that for patients infected with certain strains, combination therapy may be more impactful on clinical outcomes than for others.

### IC99 assay recapitulates findings from time-kill curves and CLSI MIC measurements

To expand on our investigation of adjunctive CFZ, we developed the 99% growth inhibition (IC99) assay, which simultaneously addresses the impracticality of time-kill curves for use in a clinical lab setting (43) and the lack of established CLSI protocols for most antibiotic combination testing (38, 44). In brief (see more details in Materials and Methods), this is a 384-well plate-based assay in which growth inhibition of an isolate is calculated from OD_600_ measurements for a series of 2-fold DAP dilutions above and below the CLSI breakpoint, either for DAP alone or with an adjunctive antibiotic at constant concentration (the adjunctive antibiotic is also tested alone at this same concentration). This allows the determination of the minimum DAP concentration required to reach 99% growth inhibition (IC99), either alone or in combination with an adjunctive antibiotic. If the isolate being tested is resistant to the adjunctive antibiotic alone, the concentration of adjunctive antibiotic used is the CLSI sensitivity breakpoint for that antibiotic; otherwise, it was the highest 2-fold dilution below the CLSI breakpoint that resulted in <98% growth inhibition.

Using the IC99 assay, we tested several DAP-R MRSA isolates, including CB5012, CB1634, and CB5036, with DAP alone and in pairwise combination with the adjunctive antibiotics CPT, CFZ, LZD, DOX, and SXT. We then replaced DAP with VAN and tested VAN alone and in combination with CFZ. Additionally, we tested the DAP-S strains CB5011, CB1631, and CB5035 (the isogenic counterparts to CB5012, CB1634, and CB5036, respectively) with DAP alone and in pairwise combination with CFZ and CPT, and VAN alone and in pairwise combination with CFZ. The DAP and VAN IC99s, alone and in each combination, and the fold change of each combination relative to DAP or VAN alone, are shown in Table 1. The concentration of each adjunctive antibiotic used and the mean ± SEM for growth inhibition by that antibiotic alone, for each isolate, are shown in Table S1. A 2-fold variation in MIC between replicates is considered an acceptable level of replicate-to-replicate variation for MIC testing (38). Therefore, we reasoned that a ≥4-fold reduction in DAP (or VAN) IC99 upon addition of an adjunctive antibiotic was required to categorize the combination as beneficial (compared to DAP or VAN alone, respectively) *in vitro* (these combinations are bold-faced in Table 1).

**TABLE 1:** For all clinical MRSA isolates used in this study, this table lists the isolate name, MprF mutations if present, and DAP and VAN IC99 values, alone and in pairwise combination with CFZ, and (for DAP) CPT, SXT, LZD, and DOX^*a*^

MRSA isolate (DAP susceptibility)	MprF mutations	DAP IC99 in µg/mL (fold-decrease)						VAN IC99 in µg/mL (fold-decrease)	
		DAP alone	DAP + CFZ	DAP + CPT	DAP + SXT	DAP + LZD	DAP + DOX	VAN alone	VAN + CFZ
CB5011 (DAP-S)	WT	0.33 ± 0.083	0.25	0.21 ± 0.042	N.T.			2.0	1.0
			(1.3 ± 0.33)	(1.7 ± 0.33)					(2)
CB5012 (DAP-R)	L826F (cytosolic)	2.4 ± 0.4	**0.75 ± 0.16**	(1)	1.3 ± 0.33	8	8	2.0	**0.5**
			(**4.0 ± 1.1**)	2.5 ± 0.5	(2)	(0.33 ± 0.083)	(0.33 ± 0.083)		(4)
CB1631 (DAP-S)	WT	0.83 ± 0.17	0.25	0.25	N.T.			3.3 ± 0.67	1.0
			(3.3 ± 0.67)	(3.3 ± 0.67)					(3.3 ± 0.67)
CB1634 (DAP-R)	L826F (cytosolic)	2.8 ± 0.49	**0.20 ± 0.031**	**0.18 ± 0.031**	1.7 ± 0.33	4.7 ± 1.8	1.7 ± 0.33	4.0	**1.2 ± 0.45**
			(**18 ± 5.9**)	(**19 ± 5.4**)	(1.3 ± 0.33)	(0.58 ± 0.22)	(1.3 ± 0.33)		(**4.7 ± 1.8**)
CB5035 (DAP-S)	WT	0.5	0.25	0.21 ± 0.042	N.T.			2	1.3 ± 0.33
			(2)	(2.7 ± 0.67)					(1.7 ± 0.33)
CB5036 (DAP-R)	P314L (other)	2	1	0.7 ± 0.12	1.3 ± 0.33	2	2.1 ± 1.1	3.3 ± 0.67	1.3 ± 0.33
			(2)	(3.0 ± 0.49)	(1.7 ± 0.33)	(1)	(3.2 ± 2.4)		(2.7 ± 0.67)
CB5013 (DAP-S)	WT	0.5	0.42 ± 0.083	0.21 ± 0.042	N.T.			2	1
			(1.3 ± 0.33)	(2.7 ± 0.67)					(2)
CB5014 (DAP-R)	S377L (other)	2.7 ± 0.67	1.7 ± 0.33	1	1.7 ± 0.33	2.7 ± 0.67	**0.77 ± 0.62**	2	1
			(1.7 ± 0.33)	(2.7 ± 0.67)	(1.7 ± 0.33)	(1)	(**16 ± 9.0**)		(2)
KPD80 (DAP-R)	L341S (other)	2	**0.42 ± 0.083**	0.83 ± 0.17	1.2 ± 0.44	3.0 ± 0.58	2	2	0.67 ± 0.17
			(**5.0 ± 1.3**)	(2.7 ± 0.67)	(2.3 ± 0.88)	(0.75 ± 0.14)	(1)		(3.3 ± 0.67)
KPD86 (DAP-R)	S337L (other)	3.5 ± 1.5	2	1.3 ± 0.33	1	6.0 ± 1.2	6.7 ± 1.3	4	2
			(2.0 ± 1.0)	(2.7 ± 0.67)	(2)	(0.56 ± 0.16)	(0.58 ± 0.22)		(2)
KPD99 (DAP-R)	P314L (other)	2	1	0.67 ± 0.17	1.5 ± 0.29	2	2	4	**1**
			(2)	(3.3 ± 0.67)	(1.5 ± 0.29)	(1)	(1)		(4)
KPD117 (DAP-R)	Q692E (cytosolic)	3.3 ± 0.67	**0.29 ± 0.11**	1.2 ± 0.44	1.8 ± 1.1	3.3 ± 0.67	6.7 ± 1.3	2	**0.5**
			(**13 ± 2.7**)	(3.3 ± 0.67)	(3.0 ± 1.0)	(1)	(0.5)		(4)
KPD113 (DAP-S)	Q692E (cytosolic)	0.5	**0.125**	N.T.					
			(4)						

^*a*^ The fold-decrease in DAP or VAN IC99 for each combination, compared with DAP or VAN alone, respectively, is also shown in parentheses. The DAP susceptibility (R = resistant, S = susceptible, by MIC testing) is listed for each isolate below the isolate number, along with any point mutations in MprF. Bold-faced indicates an *in vitro* benefit. A designation of “cytosolic” next to the mutation name indicates MprF mutations in the cytosolic C-terminal region of the synthase domain, while a designation of “other” next to the mutation name indicates other MprF synthase domain mutations. N.T. = not tested.

Adding CPT resulted in decreasing the mean DAP IC99 to ≤1 µg/mL for six of the eight DAP-R isolates tested. For one of these six isolates, CB5012, this involved a ≥4-fold decrease in DAP IC99 (of DAP + CPT compared with DAP alone) (Table 1). Adding CFZ also resulted in decreasing the mean DAP IC99 to ≤1 µg/mL for six of the eight DAP-R isolates tested; however, this involved a ≥4-fold decrease in DAP IC99 for four of those six isolates (Table 1). None of these DAP-R strains were VAN resistant (IC99 > 4 µg/mL VAN alone), but the addition of CFZ decreased the VAN IC99 ≥4-fold for four of the eight DAP-R isolates tested (Table 1). In contrast, when DOX, LZD, and SXT were tested with DAP against the eight DAP-R isolates, only the addition of DOX to DAP for one isolate (CB5014) and the addition of SXT to DAP for another isolate (KPD86) resulted in decreasing the mean DAP IC99 to 1 µg/mL. Only for CB5014 did the addition of DOX to DAP result in a ≥4-fold decrease in DAP IC99.

**Fig 2:**
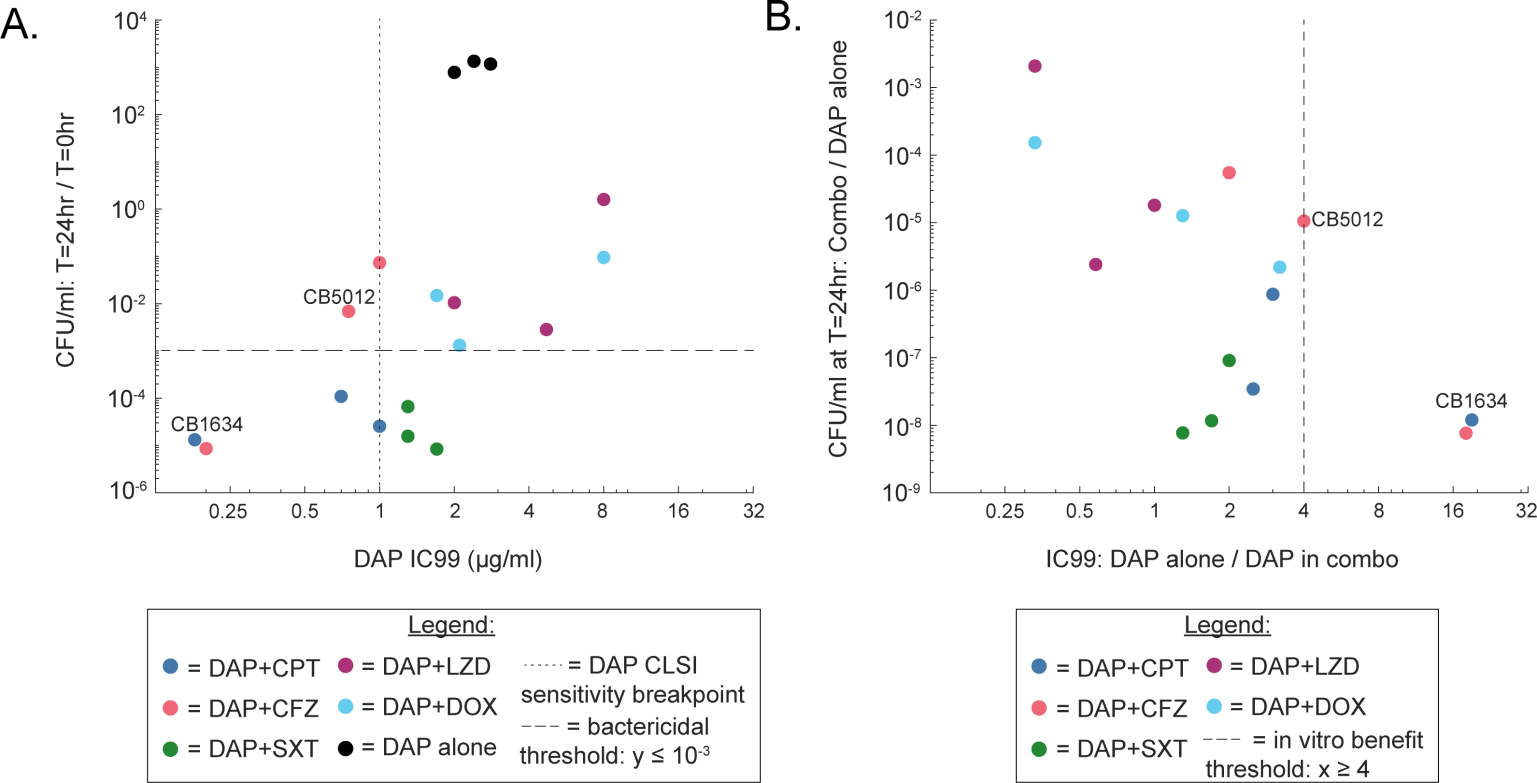
IC99 assays recapitulate results from time-kill curves. (**A**) Scatterplot where each x value represents the mean DAP IC99 value for DAP alone and in pairwise combination with CPT, CFZ, SXT, LZD, or DOX, tested against MRSA CB5012, CB1634, and CB5036; and each y value represents the CFU/mL at 24 h vs 0 h for the corresponding isolate and combination tested by time-kill assay. The Pearson correlation (R) between the log_2_-transformed x-values and log_10_-transformed y-values is r = 0.54 (*P* = 0.021). (**B**) Scatterplot where each x value is the DAP IC99 alone divided by the DAP IC99 when DAP is used in pairwise combination with CPT, CFZ, SXT, LZD, or DOX, against CB5012, CB1634, and CB5036. Each y value is the CFU/mL at 24 h of DAP in the corresponding combination, divided by the CFU/mL of DAP alone. The Pearson correlation (R) between the log_2_-transformed x-values and log_10_-transformed y-values is r = −0.66 (*P* = 0.0080).

The three best combinations for the IC99 assays were DAP + CFZ and DAP + CPT for CB1634, and DAP + CFZ for CB5012. In these three combinations, the DAP IC99 in combination was <1 µg/mL, with ≥4-fold decrease in DAP IC99 upon the addition of CPT or CFZ (Fig. 2). These combinations were synergistic and (except for one replicate for DAP + CFZ against CB5012) bactericidal in the time-kill assays.

We found that DAP mean IC99 values were within 2-fold of reported DAP MIC values for all isolates tested, and VAN mean IC99 values were within 2-fold of reported VAN MIC values for all but two of the isolates tested (Table S1). The lack of benefit of adding LZD to DAP is in agreement with some previous studies documenting antagonism with this combination against some MRSA isolates (45–47). Furthermore, our IC99 assays recapitulated our observations from the time-kill curves (Fig. 2). Lower DAP IC99 values generally corresponded to stronger bactericidal activity in the time-kill curves (Fig. 2A). Generally, for a given isolate, the better that a specific combination performed relative to DAP alone based on the time-kill curves (as measured by CFU/mL at 24 h), the better that combination performed relative to DAP alone based on the IC99 assay (DAP IC99 in combination vs DAP alone) (Fig. 2B). Specifically, we observed that the decreased DAP IC99 in combination with CFZ and CPT compared with DAP alone was recapitulated by parallel CFU/mL counts at the T = 24-h timepoint (Fig. 2B). Thus, for the isolates and antibiotics tested in this study, our IC99 assay effectively recapitulates single drug CLSI protocol MIC measurements, previous studies, as well as combination assessment done with time-kill curves.

### Use of the IC99 assay to characterize isolate variability in response to combinations

**Fig 3:**
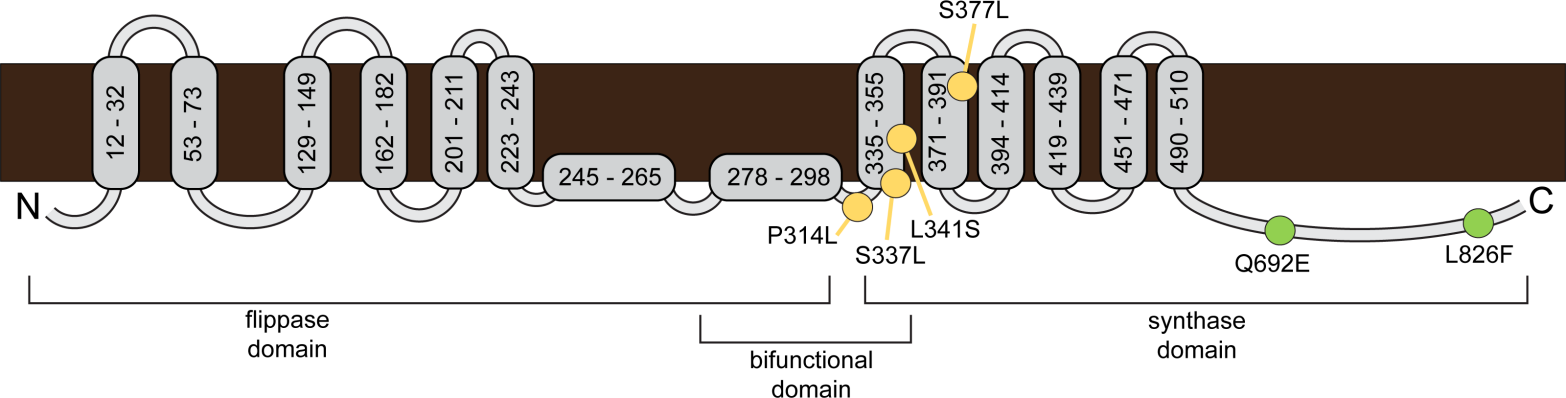
MprF mutations in the C-terminal region of the MprF synthase domain are found in isolates that show *in vitro* benefit of adding CFZ to DAP and to VAN. Diagram of the MprF protein. Membrane topology was adapted from reference 38, and domain designations were based on references 38 and 40. Mutations in MRSA isolates in this study are marked with circles; green circles indicate the mutation is found in isolate(s) that show an *in vitro* benefit when CFZ is added to DAP and to VAN. Other mutations are shown by yellow circles.

Our time-kill curve and IC99 assay results demonstrate that addition of CFZ to DAP restored DAP susceptibility was synergistic. However, there was considerable variation in the effect of CFZ addition to DAP, particularly against DAP-R isolates, which showed a mean fold decrease in DAP IC99 upon CFZ addition that ranged from <2 to 18 (Table 1). For DAP-S isolates CB5011 and CB1631, addition of CFZ to DAP resulted in <4-fold decrease in DAP IC99, but for their corresponding isogenic DAP-R isolates CB5012 and CB1634, respectively, addition of CFZ to DAP resulted in a ≥4-fold mean decrease in DAP IC99 (Table 1). In contrast, for the DAP-S/DAP-R isogenic isolate pairs CB5035 and CB5036, and CB5013 and CB5014, addition of CFZ to DAP resulted in a ≤2-fold mean decrease in DAP IC99 (Table 1). CB5012 and CB1634 both acquired the L826F mutation in MprF, which was not present in their DAP-S isogenic counterparts. In contrast, CB5036 acquired the P314L MprF mutation and CB5014 acquired the S377L MprF mutation, neither of which was present in their DAP-S counterparts (Table 1; Fig. 3). Sequencing MprF in the other tested DAP-R isolates revealed an L341S mutation in KPD80 MprF, an S337L mutation in KPD86 MprF, a P314L mutation in KPD99 MprF, and a Q692E mutation in KPD117 MprF (Table 1; Fig. 3). Interestingly, an unrelated DAP-S strain (KPD113) with Q692E MprF also showed a 4-fold mean decrease in DAP IC99 upon addition of CFZ (Table 1; Fig. 3). The results for adding CFZ to VAN followed a similar pattern: a 4-fold decrease in VAN IC99 when CFZ was added, for DAP-R isolates with the L826F or Q692E MprF mutations, but isolates without these mutations had a <4-fold decrease in VAN IC99 when CFZ was added (Table 1). Taken together, these results prompted us to hypothesize that mutations in the C-terminal cytoplasmic region of the MprF synthase domain (L826F and Q692E) could predispose a MRSA isolate to show high *in vitro* benefit when CFZ is added to DAP or VAN (Fig. 3).

### Mutations in the C-terminal cytoplasmic region of the MprF synthase domain are associated with *in vitro* benefit of adding CFZ to DAP and to VAN

**Fig 4:**
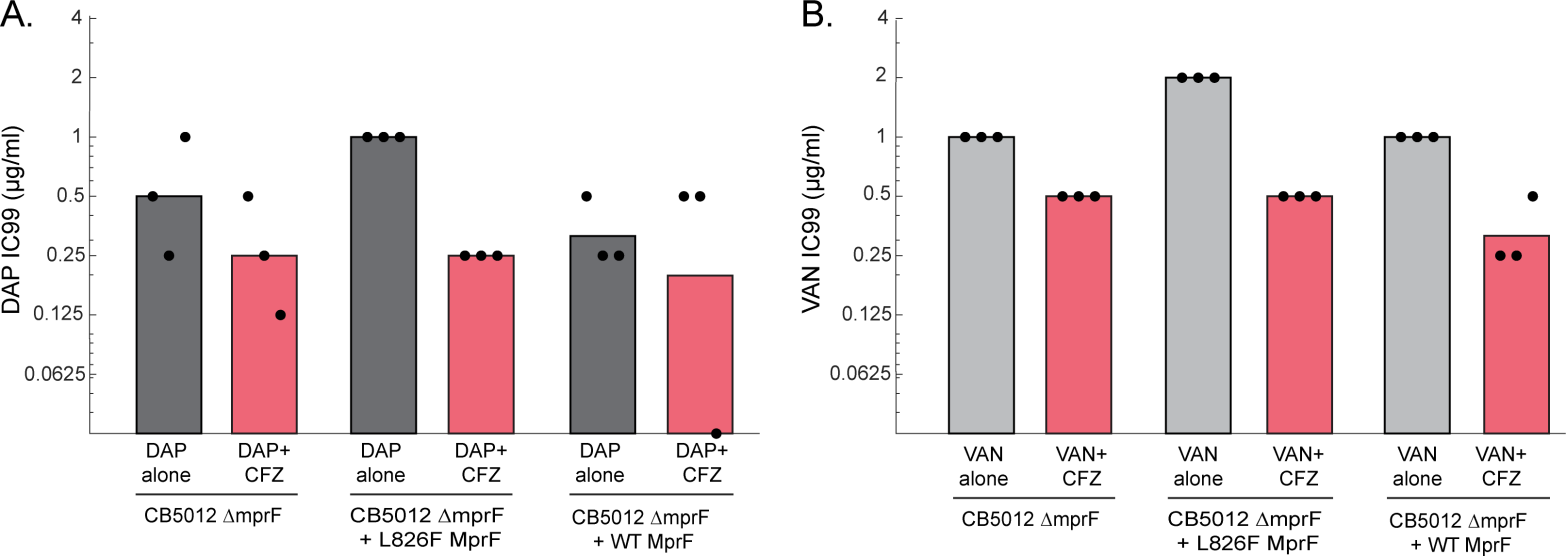
MprF deletion and complementation with L826F MprF results in a strain which shows *in vitro* benefit when CFZ is added to DAP or VAN. Graph showing the DAP IC99 (**A**) or VAN IC99 (**B**) when DAP or VAN is added alone or in combination with CFZ to CB5012 ∆mprF, CB5012 ∆mprF complemented with L826F MprF, and CB5012 ∆mprF complemented with WT MprF. Bars represent the geometric mean, and dots represent the individual results of three biological replicates for each strain and treatment group.

To test this hypothesis, we used three previously genetically constructed strains—MRSA CB5012 with the MprF gene deleted (i.e., ∆mprF), CB5012 ∆mprF transcomplemented with WT MprF, and CB5012 ∆mprF transcomplemented with L826F MprF (34). For each of the three strains, we tested DAP IC99 and VAN IC99, both alone and in combination with CFZ (Fig. 4). CFZ was used at the highest possible dose that achieved consistent results and less than full growth inhibition: 0.25 µg/mL for CB5012 ∆mprF and 1 µg/mL for other strains and combinations, except for CB5012 ∆mprF + WT MprF with VAN in CAMHB, where it was used at 0.5 µg/mL. For combination treatments, DAP and VAN IC99 were calculated by normalizing to OD_600_ of CFZ alone, instead of normalizing to untreated OD_600_, to allow for direct comparisons between all three strains, which had different levels of growth inhibition by CFZ alone. This way, the change in IC99 would be a reflection of the CFZ potentiation of DAP or VAN and not include growth inhibition by CFZ alone.

As was seen previously with these strains (34), the deletion of MprF restored DAP susceptibility (geometric mean DAP alone IC99 = 0.5 µg/mL for CB5012 ∆mprF). Transcomplementation with WT MprF maintained the DAP susceptibility (geometric mean DAP alone IC99 = 0.25 µg/mL for CB5012 ∆mprF + WT MprF), whereas transcomplementation with L826F MprF decreased DAP susceptibility (geometric mean DAP alone IC99 = 1 µg/mL for CB5012 ∆mprF + L826F MprF). For CB5012 ∆mprF, adding CFZ to DAP (Fig. 4A) and to VAN (Fig. 4B) resulted in an approximately 2-fold decrease in DAP and VAN IC99 values. For CB5012 ∆mprF + L826F MprF, adding CFZ to DAP or to VAN results in a 4-fold decrease in both DAP (Fig. 4A) and VAN (Fig. 4B) IC99 but less than 4-fold decrease for CB5012 ∆mprF + WT MprF when CFZ was added to DAP or VAN. Thus, adding CFZ to DAP or VAN for the L826F transcomplementation strain results in an *in vitro* benefit (≥4-fold decrease in IC99), while this is not the case for adding CFZ to DAP or VAN for the WT transcomplementation strain. This pattern of fold changes in DAP and VAN IC99 upon addition of CFZ is similar to what we see for MRSA isolate CB5012 (L826F MprF) compared with CB5011 (WT MprF).

## DISCUSSION

In this work, we developed a high-throughput assay referred to as the IC99 assay that recapitulated our time-kill curve results and previous single drug MIC measurements. The lower time, space, and materials cost of the IC99 assay compared with other *in vitro* combination testing methods made it possible to efficiently test additional isolates and combinations and investigate the genetic basis of combination effectiveness. A recent post-hoc study with MRSA isolates from the CAMERA2 trial found that synergistic or additive *in vitro* interactions were associated with lower 14-day patient mortality (48), which is evidence indicating the importance of strain-to-strain variability in response to drug combinations, and the relevance of *in vitro* drug interaction measurements to clinical outcomes. While the IC99 assay would be challenging to implement directly in a clinical lab setting, this study demonstrates the value of the IC99 assay for testing combinations and isolates at scale, particularly if remaining limitations can be effectively addressed. Most importantly, clinical isolates must be tested with the same treatment regimens received by the patient from which those isolates were obtained, and the results must be compared with the patient’s data to determine if the IC99 assay results can predict clinical outcomes such as mortality, duration of bacteremia, or changes in biomarkers such as procalcitonin (49) or interleukin-10 (50, 51). This would also enable us to modify the IC99 assay to use a >4-fold decrease in IC99 as the cutoff of *in vitro* benefit, if we see evidence in support of this.

Initial development of the IC99 assay, and results presented in this work, were done using growth conditions specified by CLSI MIC testing protocol, given the role of these guidelines as standards for clinical labs. However, it is likely that IC99 testing in growth medium with bicarbonate present, to mimic physiologic conditions, would better reflect clinical outcomes, since some MRSA demonstrate a “bicarbonate-response” phenotype, which alters their β-lactam susceptibility and causes them to behave as MSSA in the presence of bicarbonate (52–57). Addition of bicarbonate or other growth medium adjustments, as well as assay modifications to specifically measure cell viability and resistance development, are probable areas of exploration for future work with the IC99 assay. Such modifications would facilitate the use of the IC99 assay applications, such as further investigation of the β-lactam + DAP approach (as opposed to VAN or DAP monotherapy) for endovascular infections, particularly endocarditis. This would include assessment of the impact of β-lactam choice and MRSA genetic features on the extent of increased combination killing (compared with DAP monotherapy) and the prevention of resistance development (as can be seen during DAP monotherapy) (19, 58–60).

In this work, we used the IC99 assay (along with time-kill curves) to investigate how adding CFZ with DAP can re-sensitize DAP-resistant (DAP-R) MRSA bacteremia clinical isolates. While this study has not demonstrated that the C-terminal region of MprF is essential for seeing *in vitro* benefit when adding CFZ to DAP or VAN, the observed benefit of these combinations correlates with the presence of such mutations for the isolates we tested, and there appears to be an association at least for the isolate CB5012, based on testing the WT and mutant MprF complementation strains for this isolate (Fig. 4). This suggests that C-terminal MprF mutations may be one possible avenue by which synergy can be achieved, but fully delineating the mechanism underlying this avenue would require testing additional non-C-terminal point mutations throughout MprF, as well as testing strains with C-terminal mutations of interest (e.g., L826F and MprF) in different genetic backgrounds. In this study, CB1634 has a premature stop at position 159 in the WalK protein, another mutation associated with DAP resistance (35), and shows a much greater benefit from the addition of CFZ to DAP than does CB5012 (18-fold vs 4-fold decrease in DAP IC99). This suggests the possibility that some additional mutations besides the C-terminal MprF mutations can affect the benefit of adding CFZ to DAP, but it remains to be seen whether other additional mutations can negate the benefit of adding CFZ to DAP, even if the L826F mutation is present. For example, *cls* mutations affecting cardiolipin synthesis are more commonly found associated with MprF polymorphisms (61, 62). Thus, it would be informative to test isolates with *cls* mutations known to affect cardiolipin biosynthesis that also contain MprF polymorphisms to investigate how both mutations together affect the response to CFZ+DAP and CFZ+VAN.

Our results are not inconsistent with previous investigations. While many of the reported MprF mutations associated with DAP and/or VAN resistance are in the N-terminal half of the MprF synthase domain, one study that collected DAP-S and DAP-R isolates pre- and post-DAP treatment, respectively, identified the L776S MprF mutation and found an increased cell wall thickness for this DAP-R mutant (63). Another study identified the T646A MprF mutation in a DAP-R isolate with reduced VAN susceptibility (MIC = 4 µg/mL); this mutation did not show increased cell wall thickness but did show increased cytochrome c uptake (a proxy for cell surface charge) and increased lysyl-phosphatidylglycerol content relative to its parent DAP-S strain (64). Furthermore, the contribution of the Q692E and L826F mutations is likely not directly linked to their contributions to DAP resistance or VAN heteroresistance. The L826F mutation has been implicated in DAP resistance in a wide range of MRSA clinical isolates (31) but has also been found in DAP-S isolates (65). At least one study found a strong likelihood of association between Q692E and VAN resistance or heteroresistance (66), though another study found the Q692E mutation only among DAP-S isolates, and within this study, it was not associated with membrane phospholipid or cell surface charge changes (31). In the context of the CB1634 background, the L826F mutation results in decreased PBP2a levels due to a decrease in membrane-anchored during exposure to DAP plus the β-lactam oxacillin (20). This suggests a possible mechanism by which L826F and potentially other C-terminal MprF mutations (such as Q692E in the DAP-S isolate KPD113) could contribute to the *in vitro* benefit of adding CFZ to DAP or VAN, in a way not completely linked to DAP resistance.

## MATERIALS AND METHODS

### Clinical isolates, bacterial strains, and growth media used in this study

Thirteen MRSA clinical isolates were used in this study (Table 1; Table S1). Isogenic pairs CB5011/CB5012, CB1631/CB1634, CB5035/CB5036, and CB5013/CB5014 were a generous gift from Adriana Rosato and have been previously characterized (19, 20, 22, 34–36). Isolates KPD80, KPD86, KPD99, KPD117, and KPD113 were collected from patients at Tufts Medical Center; isolates KPD80, KPD86, and KPD99 have been tested previously (42). The MprF deletion strain made from CB5012, as well as the transcomplementation strains with WT MprF and with L826F MprF, were a generous gift from Adriana Rosato; their construction and characterization have been described previously (34). For all antibiotic testing, strains and isolates were grown in Cation-Adjusted Mueller Hinton II Broth (CAMHB), which was supplemented to a total of 50 µg/mL Ca^2+^ for any testing involving daptomycin alone or in combinations (37).

### Time-kill assays

Cultures were grown (at 37°C with shaking) overnight to saturation, then diluted back to OD_600_ = 0.001 (~10^6^ CFU/mL), and aliquoted into the non-edge wells of 96-well plates (300 μL/well). Prior to aliquoting, an HP D300E digital dispenser was used to dispense antibiotic stock solutions into the wells. Antibiotics tested included DAP (1 µg/mL), CFZ (2 µg/mL), CPT (1 µg/mL), SXT (2/38 µg/mL trimethoprim-sulfamethoxazole), LZD (4 µg/mL), DOX (4 µg/mL), as well as DAP in combination with each of the other antibiotics. Positive control wells were left untreated, and 300 μL sterile CAMHB was added to edge wells of the 96-well plates. CFU counts were taken by plating serial dilutions onto CAMHB agar plates; CFU counts were done at 0, 2, 4, 8, and 24 h for DAP combinations with CFZ, CPT, and SXT, and 0, 16, and 24 h for DAP combinations with LZD and DOX. We used additional and more closely spaced early time points for DAP in pairwise combination with CFZ, SXT, and CPT, compared with DAP in pairwise combination with LZD and DOX, so we could effectively compare the combinations and the component single drugs across strains, even with the greater and faster killing observed for these single and pairwise combinations in some strains. Results presented are mean ± standard error of the mean for 2–3 biological replicates.

### IC99 assays

Cultures were grown (at 37°C with shaking) overnight to saturation, then diluted back to OD_600_ = 0.001 (10^6^ CFU/mL), and aliquoted into the non-edge wells of 384-well plates (50 µL/well). Prior to aliquoting, an HP D300E digital dispenser was used to dispense antibiotic stock solutions into the wells. For all DAP-R isolates (CB5012, CB1634, CB5036, CB5014, KPD80, KPD86, KPD99, KPD117), DAP was tested alone and in combination with CFZ, CPT, SXT, LZD, and DOX, and VAN was tested alone and in combination with CFZ. For DAP-S isolates CB5011, CB1631, CB5035, and CB5013, DAP was tested alone and with CFZ and CPT, and VAN was tested alone and with CFZ. For DAP-S isolate KPD113, DAP was tested alone and with CFZ. For all IC99 assays, DAP and VAN were tested at a series of 2-fold dilutions ranging from above and below the CLSI sensitivity breakpoint (e.g., 1×, 2× MIC values). When an adjunctive antibiotic was added (CFZ, CPT, SXT, LZD, or DOX), it was used at a constant concentration. This concentration was either the antibiotic’s CLSI sensitivity breakpoint for *S. aureus* if the isolate being tested was resistant to the adjunctive antibiotic, and if the isolate being tested was sensitive to the adjunctive antibiotic, the concentration used was the highest 2-fold dilution below the sensitivity breakpoint that resulted in <98% growth inhibition (when the adjunctive antibiotic is used alone). These concentrations and growth inhibition by the adjunctive antibiotics alone are listed in Table S1. The 384-well plates were allowed to grow for 16–20 h or for 24 h when VAN was tested alone and with CFZ, and then OD_600_ measurements were made with a Biotek HT plate reader. For each well treated with antibiotic(s), growth inhibition was calculated using the formula:


$$Growth\;Inhibition=1-\left(\frac{treated\;well\;OD_{600}-background\;OD_{600}\;}{untreated\;OD_{600}-background\;OD_{600}}\right)$$

In this formula, background OD_600_ is the median OD_600_ of the edge wells of the 384-well plate, which contained sterile media. Untreated OD_600_ is the median OD_600_ all non-edge wells on the plate that contained culture but no antibiotics. For DAP alone and in combination with each adjunctive antibiotic (and for VAN alone and in combination with CFZ), the DAP (or VAN) IC99 was calculated as the lowest tested concentration of DAP (or VAN) required to reach a growth inhibition of 0.99, i.e., 99% growth inhibition. For DAP combinations, the fold change between the DAP IC99 of the combination and the DAP alone IC99 was calculated; the same was done for VAN in combination with CFZ. Results presented are mean ± standard error of the mean for at least three biological replicates.

## SUPPLEMENTAL MATERIAL

Supplemental material aac.01547-25-s0001.docx Fig. S1; Table S1.

ASM does not own the copyrights to Supplemental Material that may be linked to, or accessed through, an article. The authors have granted ASM a non-exclusive, world-wide license to publish the Supplemental Material files. Please contact the corresponding author directly for reuse.
