# Supplementary material for: Restoration of daptomycin sensitivity with adjunctive cefazolin is associated with C-terminal MprF mutations in MRSA bacteremia isolates
Source: Antimicrob Agents Chemother. 2026 Apr 27;70(6):e01547-25. doi: 10.1128/aac.01547-25 (PMC13231919; doi:10.1128/aac.01547-25)
Supplement: Supplemental material — Fig. S1; Table S1. [file aac.01547-25-s0001.docx]

**Supplemental Tables and Figures**


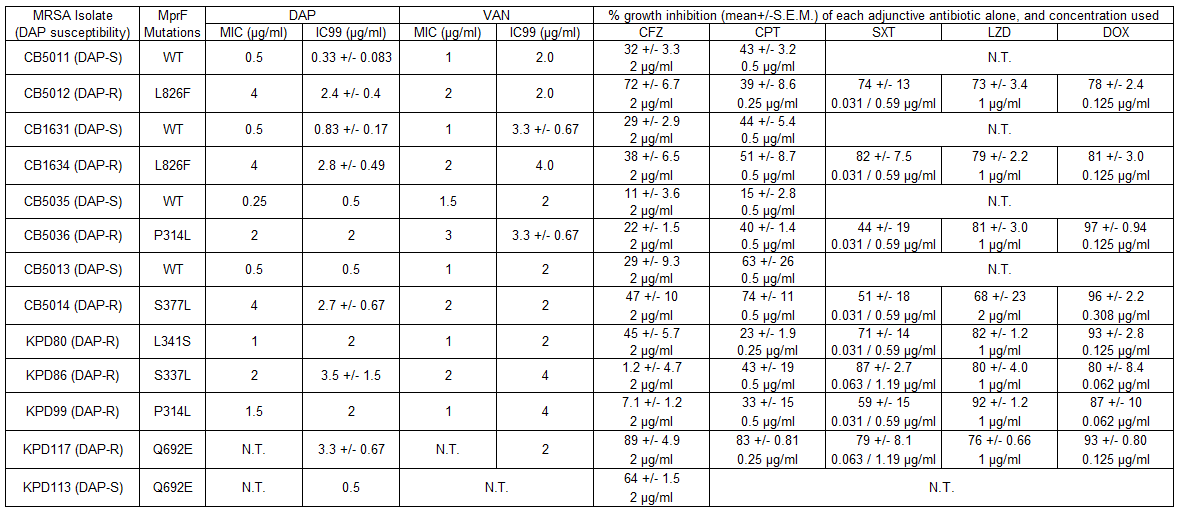


Table S1: For all clinical MRSA isolates used in this study, this table lists the isolate name, MprF mutations if present, and previously reported DAP and VAN MIC values. IC99 values for DAP alone and VAN alone measured in this study (from Table 1) are also shown here for comparison. For each isolate, the concentration of each adjunctive antibiotic used (both alone and in pairwise combinations) is listed, along with the percent growth inhibition when that adjunctive antibiotic is used alone (value given is mean + / - standard error of the mean for at least three biological replicates).


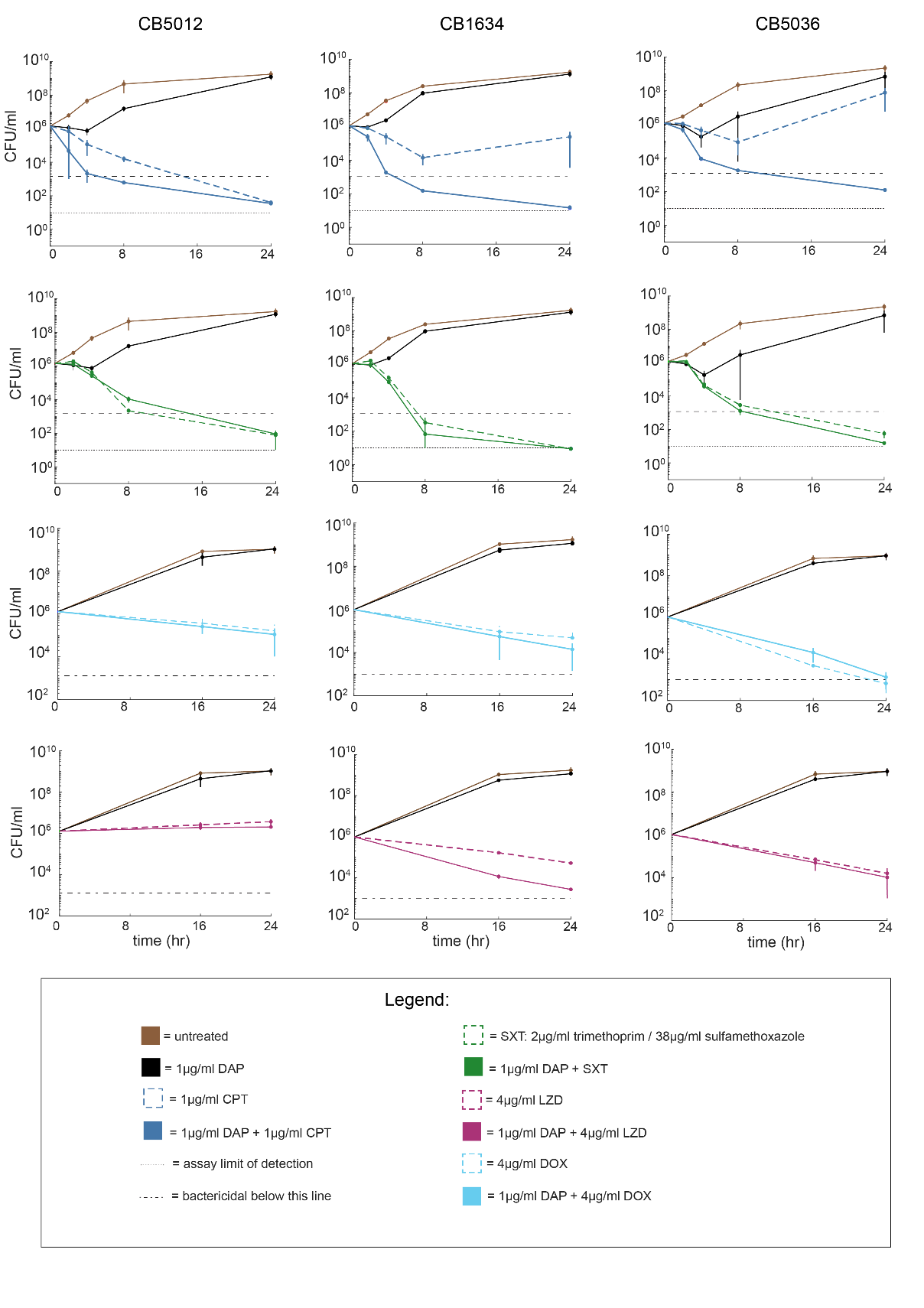


**Figure S1: Time-kill curves for DAP alone and in pairwise combinations.** Replicates of three different daptomycin-resistant MRSA isolates: CB5012, CB1634, and CB5036 (left column to right column) treated with 1µg/ml DAP alone (black) or in pairwise combination with (top row to bottom row) 1µg/ml CPT (blue), 2 / 38 µg/ml SXT (green), 4µg/ml DOX (light blue), or 4µg/ml LZD (purple). Each adjunctive antibiotic was also tested alone (dashed lines of the same color), and also compared to untreated (brown). The starting inoculum was targeted to 10^6^ CFU/ml, and CFU/ml quantifications were done at 2hr, 4hr, 8hr, and 24hr post-treatment for DAP + CPT, DAP + CFZ, and DAP + SXT, and at 16hr and 24hr post-treatment for DAP + DOX and DAP + LZD. Results shown are mean +/- standard error of the mean for at least three biological replicates. The assay limit of detection is shown where appropriate (1-10 CFU/ml, horizontal dotted black line), and the criteria for bactericidality (3 log_10_ fold decrease from starting CFU/ml, horizontal dashed black line) is also shown.
